# Pharmacokinetics, pharmacodynamics, and safety of ciprofol emulsion in Chinese subjects with normal or impaired renal function

**DOI:** 10.3389/fphar.2023.1260599

**Published:** 2023-11-23

**Authors:** Jun Tao, Shuaibing Liu, Ying Ying Zhao, Lei Qi, Pangke Yan, Nan Wu, Xiao Liu, Dongwei Liu, Xin Tian

**Affiliations:** ^1^ Department of Pharmacy, The First Affiliated Hospital of Zhengzhou University, Zhengzhou, China; ^2^ Henan Provincial Key Laboratory of Precision Clinical Pharmacy, Zhengzhou University, Zhengzhou, China; ^3^ Department of Anesthesiology, Pain and Perioperative Medicine, The First Affiliated Hospital of Zhengzhou University, Zhengzhou, China; ^4^ Sichuan Haisco Pharmaceutical Group Co., Ltd., Chengdu, China; ^5^ Department of Nephrology, The First Affiliated Hospital of Zhengzhou University, Zhengzhou, China

**Keywords:** pharmacokinetics, pharmacodynamics, safety, ciprofol, anesthetic, renal impairment

## Abstract

**Background:** Ciprofol, a novel sedative–hypnotic drug, has been approved for its use in inducing and maintaining general anesthesia, as well as for providing sedation.

**Methods:** In this phase I, single-center, parallel, controlled, open-label clinical trial, our objective was to analyze the pharmacokinetics (PK), pharmacodynamics (PD), and safety of ciprofol emulsion in 24 participants with mild and moderate renal impairment (*n* = 8 per group) and matched healthy participants (*n* = 8). An initial loading infusion of ciprofol was administered at 0.4 mg/kg for 1 min, followed by a maintenance infusion at a rate of 0.4 mg/kg/h for 30 min. We collected plasma and urine samples from the participants to assess the PK of ciprofol and its metabolite M4. The evaluation of PD involved using a modified observer’s alertness/sedation scale (MOAA/S) in combination with bispectral index (BIS) monitoring. Safety assessments were conducted throughout the trial process.

**Results:** The plasma concentration–time curve of ciprofol in participants with renal impairment was similar to that in participants with normal kidney function. The area under the curve (AUC) and maximum concentration (C_max_) of total and unbound ciprofol in plasma for participants with renal impairment were only slightly higher (0.7–1.2-fold) than those in participants with normal renal function. In contrast, for the metabolite M4, AUC values were 1.3- and 2.1-fold greater in participants with mild and moderate renal impairment, respectively, than in healthy controls. However, renal impairment had no significant impact on the PD parameters. The study found that ciprofol was well-tolerated, with all adverse events (AEs) reported being mild or moderate in severity.

**Conclusion:** Based on these findings, we can conclude that no dosage adjustment of ciprofol is necessary for patients with mild–moderate renal impairment who receive the injection.

**Clinical Trial Registration:** https://clinicaltrials.gov, identifier NCT04142970.

## 1 Introduction

Ciprofol (HSK3486) is a sedative–hypnotic compound approved in 2020 for use in inducing and maintaining sedation, as well as providing general anesthesia. The primary mechanism of action of ciprofol is to enhance the activity of the ion channel mediated by gamma–aminobutyric acid type A (GABA_A_) receptors, resulting in an influx of chloride ions (Qin et al., 2017). This leads to suppression of the central nervous system. This channel is also the primary target of propofol, a commonly used intravenous anesthetic with rapid onset and a short duration of action (Sahinovic et al., 2018). However, propofol has limitations, including injection pain, hemodynamic issues, and the risk of a potentially fatal condition known as propofol infusion syndrome (PIS) (Mahmoud and Mason, 2018). To address these limitations and improve the pharmacological and physicochemical properties of drug–receptor binding, propofol was optimized into ciprofol by incorporating a cyclopropyl group (Qin et al., 2017). Ciprofol has been shown to be highly effective, causing significant reductions in pain and improved hemodynamic stability (Qin et al., 2017; Li et al., 2022; Liu et al., 2022; Wang et al., 2022).

Ciprofol exhibits wide tissue distribution, with approximately 95% of the drug binding to plasma proteins (Lu et al., 2023). It is primarily metabolized in the liver by phase II glucuronosyltransferases (UGTs), and UGT1A9 is the main enzyme responsible for converting ciprofol into its major metabolite, M4. Subsequently, M4 is excreted through the renal route (84.59%) (Bian et al., 2021). The M4 metabolite does not exhibit any toxic or hypnotic properties, and it may not be necessary to evaluate the function of the kidney in removing this metabolite (Bian et al., 2021). It is well-established that renal impairment can have a notable effect on the renal excretion of drugs, as well as their metabolism and transport in the kidney, liver, and intestine. Additionally, it can impact the protein binding of drugs, resulting in substantial changes in both pharmacokinetics (PK) and pharmacodynamics (PD) (Boucher et al., 2006; Dixon et al., 2014; Celestin and Musteata, 2021). Thus, regulatory agencies recommend that studies be conducted to evaluate the potential effects of renal impairment on the PK and PD of any drug that may be used in patients with renal impairment, even if the kidneys are not primarily the elimination route for small molecules of drugs or their active metabolites (European Medicines Agency, 2015; U.S. Food and Drug Administration, 2020). Previous investigations have explored the PK, PD, and safety of ciprofol in a few special populations, including patients with hepatic impairment, the elderly (ages 65–73) vs. younger adults (ages 21–44), and male vs. female subgroups (Li et al., 2021; Teng et al., 2021; Hu et al., 2022). However, there were limited data to determine whether dose adjustment of ciprofol is necessary in patients with renal impairment.

In the current study, our objectives were to explore the PK profiles of total ciprofol, unbound ciprofol, and its major metabolite M4 and to evaluate the PD of ciprofol and its safety profile in patients with mild-to-moderate renal impairment, as well as in healthy controls. These results may provide essential information for recommending appropriate dosage adjustments of ciprofol for patients with varying stages of renal impairment.

## 2 Materials and methods

### 2.1 Subjects

The study, conducted at the First Affiliated Hospital of Zhengzhou University in China between November 2019 and August 2020, received approval from an independent Ethics Committee. All participants provided written informed consent. This clinical trial (NCT04142970, principal investigator: Zhangsuo Liu, date of registration: October 2019, https://clinicaltrials.gov/) adhered to ethical guidelines, such as the Declaration of Helsinki principles and Good Clinical Practice rules.

Participants eligible for the trial were males and females aged between 18 and 65, with a body weight ≥45 kg and a body mass index (BMI) ranging from 18 to 28 kg/m^2^. Exclusion criteria included individuals with any clinically significant medical conditions (except for renal impairment or its underlying causes); medical instability; including psychiatric or neurological disorders; cardiovascular, endocrine, pulmonary, hepatic, gastrointestinal, or metabolic illnesses; or any other condition that could interfere with the assessment of the PK of the investigational drug or the completion of the trial. Potential participants underwent a pre-study screening, which involved reviewing their medical history, physical examinations, a 12-lead ECG, and monitoring of vital signs (blood pressure, oxygen saturation, and respiratory and cardiac rates). Laboratory examinations, including coagulation, hematology, clinical chemistry, and urinalysis, were conducted 14 days prior to the study.

### 2.2 Study design and procedures

In this phase I, single-center, parallel, controlled, open-label clinical trial, the participants were admitted to the clinic the day before receiving medication. They fasted for at least 8 h and abstained from drinking water for at least 2 h prior to drug administration. On the subsequent morning, the participants were administered a 0.4 mg/kg dose of ciprofol as a 1-min bolus, followed by a continuous infusion of 0.4 mg/kg/h for 30 min using a pump in a fully equipped operating room. To calculate the rate of ciprofol administration, the following formulas were used: dosing rate of the loading dose (mL/h) = 0.4 mg/kg × subject’s baseline weight (kg) ÷ 2.5 mg/mL × 60 h^−1^ and administration rate of the maintenance dose (mL/h) = 0.4 mg/kg/h × baseline weight (kg) ÷ 2.5 mg/mL. The degree of anesthesia or sedation experienced by participants was evaluated using two methods: the modified observer’s alertness/sedation scale (MOAA/S) and bispectral index (BIS) monitoring. Vital signs, electrocardiograms, and other indicators were monitored using an electrocardiogram monitor. These observations continued until the participants were fully awakened and achieved an MOAA/S score of 5 for three consecutive assessments. MOAA/S scores (Yamada et al., 2022) (Supplementary Table S1) were evaluated at various time points, including 5 min before drug administration, 1 min after the start of the loading dose, every 5 min during the continuous maintenance infusion, and every 2 min until the end of the infusion. BIS values were recorded 5 min before and 60 min after drug administration. Each subject received 100% oxygen via a mask until they were fully awake. Food and water were provided to the participants after they had fully awakened following medication administration.

Arterial blood samples (3 mL in volume) were collected at specific time intervals, including 30 min before administration; 1 min after the completion of the loading infusion; and at 5, 10, 20, and 30 min after the start of the maintenance infusion, as well as at 1, 2, 4, 8, 15, 30 min, and 1 h after the end of the infusion. Venous blood samples (3 mL in volume) were collected at 2, 3, 4, 6, 8, 12, 24, and 48 h after drug administration. In addition, each 5 mL of arterial blood was sampled to estimate the protein binding rate, 1 min after the start of administration and 1 min after the end of the infusion. Blood samples were collected in K_2_EDTA-containing tubes and centrifuged for 10 min at 4°C and 1,700 × g, and the plasma was separated and stored at–80°C.

Urine samples were collected over a 24-h period before ciprofol administration and at specific time intervals after administration: 0–4, 4–8, 8–12, 12–24, and 24–48 h. The urine samples (3 mL) were aliquoted into polypropylene tubes and cryopreserved at −80°C for future analysis.

Once the final PK samples were collected and safety evaluations were completed, the participants were allowed to leave the clinical site on the third day.

### 2.3 Assay of ciprofol and M4

The concentrations of ciprofol and its metabolite M4 in plasma, as well as the concentration of M4 in urine, were evaluated using established high-performance liquid chromatography-tandem mass spectrometry (HPLC-MS/MS) techniques. The analytical instrument used for the analysis was an LC-30 AD system (Shimadzu), coupled to a Triple Quad 6500^+^ mass spectrometer and Analyst™ 1.6.3 software (both AB Sciex). Protein precipitation was performed as a pretreatment step for human plasma and urine samples. To monitor ciprofol and M4, multi-reaction monitoring was performed in negative mode. The compound HSK23287 served as the internal standard for assessing ciprofol, while nimesulide was used as the internal standard for M4. A quadratic regression analysis was conducted to determine the best-fit line for the calibration standards, with a weighing factor set to 1/x^2^. The linear range of ciprofol and M4 in plasma was 5–5,000 ng/mL, and in urine, it was 10–10,000 ng/mL.

### 2.4 Pharmacokinetic and pharmacodynamic analyses

PK parameters were computed using a noncompartmental approach with the Phoenix WinNonlin 8.3.1 (Certara, L.P., Princeton, NJ, United States). The maximum plasma concentration (C_max_) was directly obtained from the plasma concentration–time plot. The peak time (T_max_) was defined as the time at which C_max_ was obtained. The area under the plasma concentration–time curve (AUC) from 0 to t (AUC_0-t_) was computed using the linear trapezoidal rule. AUC_0-inf_ was calculated as follows: AUC_0-inf_ = AUC_0-t_ + C_t_/k_e_, where C_t_ is the final determined concentration and k_e_ is estimated by linear regression of the log-linear part of the plasma concentration–time curve. The terminal elimination half-life (t_1/2_) was calculated as ln2/ke, the total clearance (CL) as Dose/AUC_0-inf_, and the distribution volume (V_d_) as Dose/(AUC_0-inf_* k_e_). Protein binding (PB) was measured with equilibrium dialysis. For each participant, the protein binding was calculated as the average following the loading infusion and 1 min after the infusion was discontinued. The fraction unbound (Fu) was calculated as follows: Fu (%) = 100%—PB (%). Unbound concentration was estimated using the measured total concentration at each time point multiplied by Fu, assumed to be constant. The unbound C_max_ (C_max,u_) of ciprofol was calculated as C_max_ × Fu(%), and unbound AUC_0-inf_ (AUC_0-inf,u_) of ciprofol was calculated using the linear trapezoidal rule, as previously described, from the unbound ciprofol concentrations.

The PK parameters of urine were as follows: cumulative excretion (Ae_0–48_) and renal clearance (CL_R_) of M4. Ae_0–48_ was determined by summing the quantity of M4 excreted in urine between 0 and 48 h following dosing. CL_R_ was computed as Ae_0–48_/AUC_0-t_.

PD parameters were calculated using Adopt SAS^®^ 9.4 (SAS Institute, Cary, North Carolina, United States). The PD parameters included the following: minimum BIS value (BIS_peak_), the time to reach BIS_peak_ (T_BISpeak_), and BIS AUC_0-t_.

### 2.5 Safety

For safety assessment, the alterations in vital signs, ECG signals, laboratory findings, physical examinations, and assessments of injection pain were surveilled. Particular attention was given to adverse reactions associated with sedation, such as bradycardia, apnea, hypoxia, and hypotension.

### 2.6 Statistics analysis

Continuous variables were expressed as medians (maximum and minimum) or means (standard deviations). Adopt SAS^®^ 9.4 (SAS Institute, Cary, North Carolina, United States) was used to statistically assess the PK and PD data. The impact of renal impairment versus the control group was investigated using analysis of variance (ANOVA), which included renal function as a fixed effect, and the corresponding 90% confidence intervals (CIs) around the geometric least-squares mean (GLSM) ratio of C_max_, AUC_0-inf_, C_max,u_, and AUC_0-inf,u_ were calculated. Multiple regression analysis was conducted to assess the correlation between clinical variables (age, sex, weight, and eGFR) and PK parameters (C_max_, AUC_0-inf_, CL, C_max,u_, AUC_0-inf,u_, CL_u_, and CL_R_). The correlation was determined by multiple regression analysis, using PK parameters as the dependent variable and clinical variables as independent variables.

## 3 Results

### 3.1 Subject demographics

**FIGURE 1:**
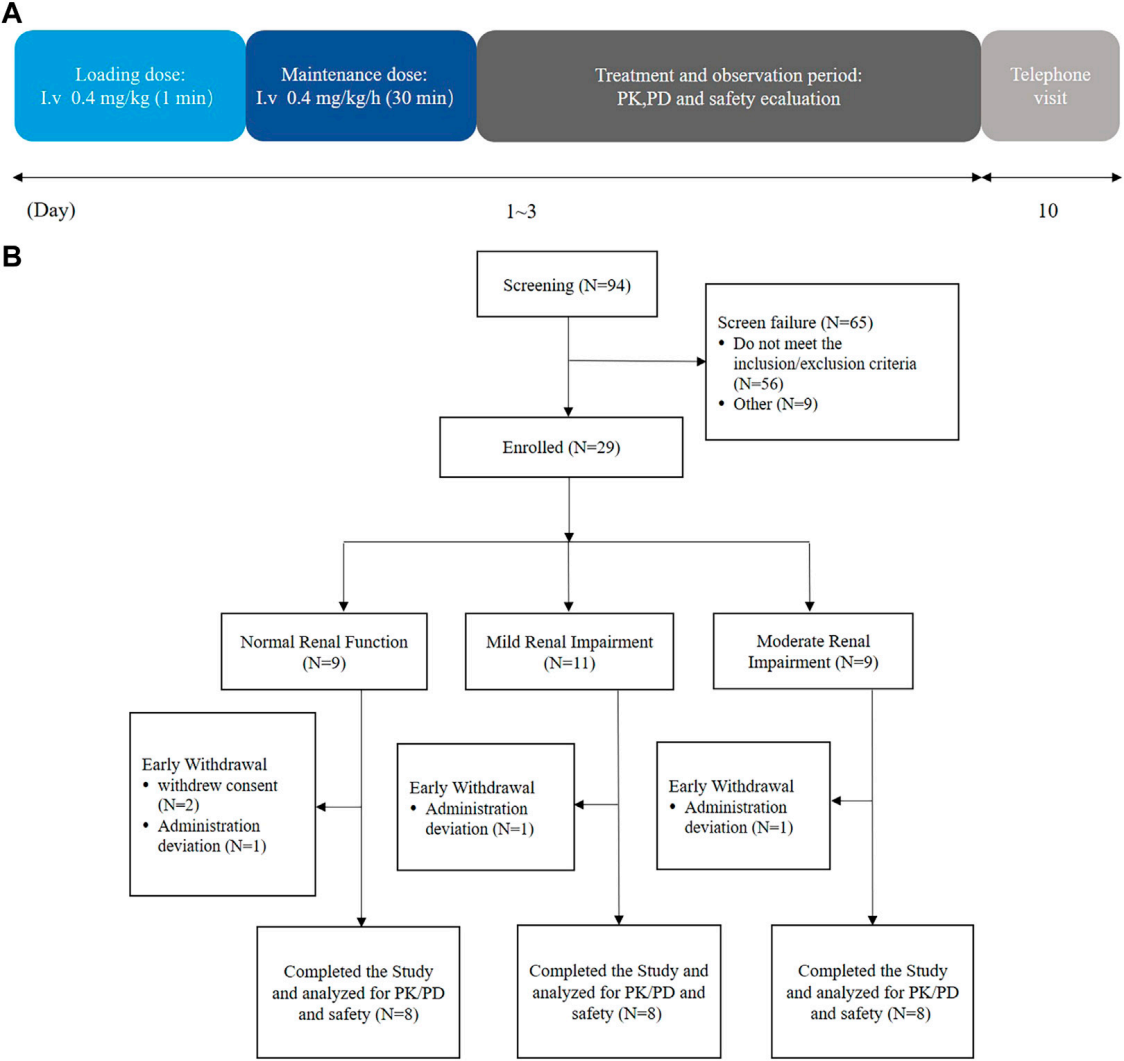
Flow diagrams of the current trial. **(A)** Schematic of the trial duration and **(B)** schematic of the trial participants.

A total of 24 participants with varying degrees of renal function, i.e., mildly impaired function (eGFR: 60–89 mL/min/1.73 m^2^), moderately impaired function (eGFR: 30–59 mL/min/1.73 m^2^), and normal kidney function (eGFR: ≥90 mL/min/1.73 m^2^) (*n* = 8 for each group), were enrolled in the study (Figure 1). The categorization of renal function was established by assessing the estimated glomerular filtration rate (eGFR) through the modification of diet in renal disease (MDRD) equation (Levey et al., 2006): eGFR (mL/min/1.73 m^2^) = 175 × (Scr, std)^−1.234^ × (age)^−0.179^ × (0.79 for females). The demographic attributes of the normal control participants closely resembled those of the participants with renal impairment (Table 1).

**TABLE 1:** Demographic and baseline characteristics.

Parameter	Renal function group		
	Normal (*n* = 8)	Mild (*n* = 8)	Moderate (*n* = 8)
Sex [M/F (n)]	6/2	7/1	6/2
Age (years)	44.1 (1.2)	41.4 (8.0)	47.8 (11.0)
Weight (kg)	63.9 (4.7)	71.0 (8.4)	60.7 (8.2)
BMI (kg/m^2^)	23.7 (1.5)	25.7 (1.5)	23.5 (2.5)
eGFR (mL/min/1.73 m^2^)	128.6 (20.7)	72.7 (7.5)	43.0 (10.6)

Unless otherwise specified, all data are reported as means (SDs). The body mass index (BMI) is formulated as [weight in kg ÷ (height in m)^2^]. The modification of diet in renal disease (MDRD) equation is used to compute the estimated glomerular filtration rate (eGFR).

### 3.2 Pharmacokinetics

**FIGURE 2:**
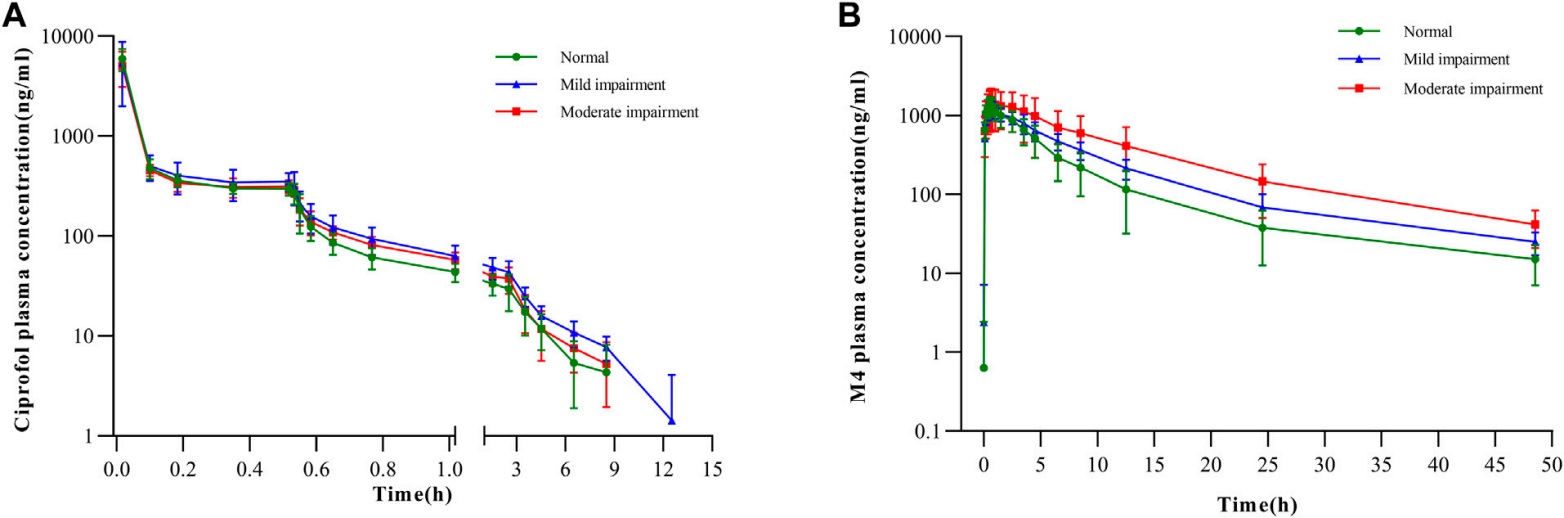
Plasma concentration–time curves for **(A)** ciprofol in semi-log scale and **(B)** M4 in semi-log scale.

The mean plasma concentration–time graphs of ciprofol and its metabolite M4 are shown in Figure 2. In Figure 2A, it is evident that the plasma concentration–time curve of ciprofol is similar across the three groups. The C_max_ of ciprofol was achieved 1 min after administration. Following the cessation of administration, the plasma concentration of ciprofol measured 352 ng/mL in the mild renal impairment group, 314 ng/mL in the moderate renal impairment group, and 297 ng/mL in the normal kidney function control group. The concentration of ciprofol in plasma decreased to the lowest limit of quantitation (5 ng/mL) approximately 8–12 h after the administration was stopped.

The primary PK parameters of ciprofol are presented in Table 2. The results showed that compared to the healthy control participants, the individuals with mild renal impairment exhibited an 8.56% decrease in C_max_ and a 15.67% increase in AUC_0-inf_ of ciprofol. On the other hand, the individuals with moderate renal impairment showed a decline of 15.15% and 1.80% in C_max_ and AUC_0-inf_ of ciprofol, respectively (Table 2, 3). The C_max_ values in the participants with mild and moderate renal impairment showed significant inter-individual variability, with coefficient of variation (CV%) values amounting to 138.15% and 42.72%, respectively.

**TABLE 2:** Pharmacokinetic (pk) parameters in plasma and urine collected from participants with normal and impaired renal function.

Parameter	Renal function group		
	Normal (*n* = 8)	Mild (*n* = 8)	Moderate (*n* = 8)
Plasma ciprofol			
C_max_ (ng/mL)	5,941.25 (1,478.36)	5,432.50 (3,314.53)	5,041.25 (1,945.57)
T_max_ (h)	0.02 (0.02, 0.02)	0.02 (0.02, 0.10)	0.02 (0.02, 0.02)
AUC_0-t_ (ng•h/mL)	521.99 (77.30)	596.24 (167.44)	517.71 (98.52)
AUC_0-inf_ (ng•h/mL)	549.18 (87.17)	635.26 (174.15)	539.31 (99.40)
CL (L/h)	70.60 (10.58)	69.54 (14.21)	68.52 (11.40)
V_d_ (L)	272.41 (99.04)	378.12 (182.56)	212.49 (95.90)
t_1/2_ (h)	2.82 (1.39)	4.02 (2.58)	2.13 (0.71)
Plasma unbound ciprofol			
C_max, u_ (ng/mL)	52.74 (11.39)	46.97 (29.51)	37.55 (12.79)
AUC_0-t,u_ (ng•h/mL)	4.67 (0.71)	5.09 (1.56)	3.91 (0.61)
AUC_0-inf,u_ (ng•h/mL)	4.91 (0.76)	5.42 (1.62)	4.07 (0.61)
CL_u_ (L/h)	7,841.62 (778.60)	8,297.30 (2,093.28)	9,023.51 (1,470.86)
V_d,u_ (L)	31,321.77 (14,232.70)	44,791.11 (20,668.31)	27,957.51 (12,240.64)
Plasma metabolite M4			
C_max_ (ng/mL)	1,371.75 (345.81)	1,250.63 (249.83)	1,455.50 (768.97)
T_max_ (h)	0.55 (0.53, 0.65)	0.62 (0.50, 1.57)	0.62 (0.53, 2.52)
AUC_0-t_ (ng•h/mL)	7,285.16 (3,010.77)	9,721.68 (2,322.06)	15,377.32 (9,426.84)
AUC_0-inf_ (ng•h/mL)	7,538.73 (3,111.54)	10,058.72 (2,438.99)	16,029.21 (9,662.07)
t_1/2_ (h)	11.71 (4.20)	9.01 (1.55)	11.04 (1.75)
Urine metabolite M4			
Ae_0-t_ (mg)	23.85 (6.92)	23.83 (4.18)	16.76 (5.18)
CL_R_	3.50 (0.84)	2.61 (0.90)	1.23 (0.39)

Data are means (SDs) for all, except time to maximum concentration (T_max_), which is the median (range).

C_max_, maximum observed concentration; AUC_0-t_, area under the curve from 0 to 48 h after the end of administration; AUC_0-inf_, area under the curve from 0 to infinity time; CL, total clearance; V_d_, distribution volume; t_1/2_, terminal elimination half-life; u, free fraction; Ae, accumulative urine excretion; CL_R_, renal clearance.

**TABLE 3:** Statistical summaries of PK parameters for participants with mild and moderate renal impairment in comparison to the normal renal function controls.

Parameter	GLSM		Ratio (%)	90% CI of ratio (%)
	Renal impairment	Normal renal function		
Mild renal impairment vs. normal renal function				
C_max_	3,933.47	5,770.81	68.16	(38.65, 120.20)
AUC_0-inf_	614.90	543.24	113.19	(94.36, 135.77)
C_max,u_	33.32	51.63	64.55	(36.52, 114.07)
AUC_0-inf,u_	5.20	4.86	107.19	(89.22, 128.79)
Moderate renal impairment vs. normal renal function				
C_max_	4,699.23	5,770.81	81.43	(46.18, 143.60)
AUC_0-inf_	531.21	543.24	97.78	(81.52, 117.29)
C_max,u_	35.65	51.63	69.05	(39.07, 122.03)
AUC_0-inf,u_	4.03	4.86	82.92	(69.01, 99.62)

Natural log-transformed parameters were subjected to analysis of variance, where groups served as the fixed effect and subjects as the random effect. For the parameters, the geometric least-squares mean (GLSM) difference and its 90% confidence interval (CI) are obtained. The inverse logarithm is used in the aforementioned findings to obtain the point estimation and 90% CI for the GLSM ratio of the parameters between the two groups.

The results of the protein binding assay indicated that ciprofol exhibits a high degree of binding to plasma proteins. The mean protein binding (%) for ciprofol was 99.10%, 99.15%, and 99.24% among the normal, mild renal impairment, and moderate renal impairment groups, respectively. The Fu (%) was 0.9%, 0.85%, and 0.76% for healthy participants and patients with mild and moderate renal impairment, respectively. As renal function worsened, decreased Fu was noted.

In comparison to participants with normal renal function, those with mild renal impairment demonstrated a 10.94% decrease in C_max, u_ and a 10.39% increase in AUC_0-inf, u_ for unbound ciprofol (Table 2). On the other hand, participants with moderate renal impairment exhibited 28.80% and 17.11% reductions in C_max, u_ and AUC_0-inf, u_, respectively, in contrast with individuals with normal renal function (Table 2, 3). Similarly, there was substantial variability in C_max, u_ within the mildly and moderately impaired groups, with CV% values amounting to 146.04% and 36.02%, respectively.

There was no apparent trend observed in C_max_, and t_1/2_ of M4 among the three groups with respect to the aggravation of renal function damage (Table 2). Nonetheless, there was an observed increase in AUC_0-inf_ with increase in the degree of kidney impairment (Figure 2B). The mean AUC_0-inf_ increased by 33.43% and 112.62% in the mildly and moderately impaired groups, respectively (Table 2). In contrast to those with normal renal function, those with moderate impairment showed a 29.73% decrease in Ae_0-t_ (Table 2). However, there was no clear difference in Ae_0-t_ between the mild renal impairment and normal function groups.

**FIGURE 3:**
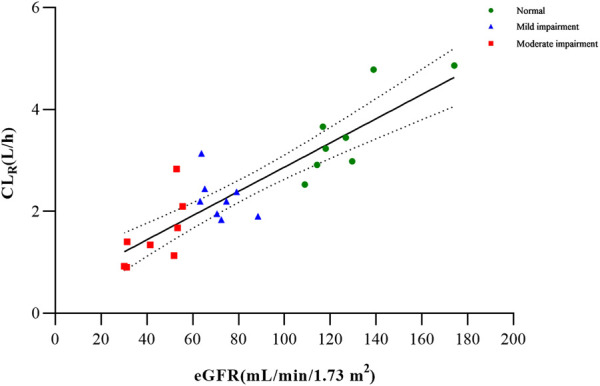
Correlation between CL_R_ and eGFR across the renal function groups (*p* < 0.0001).

Based on the findings from the multiple regression analysis conducted on PK parameters and clinical variables (age, sex, weight, and eGFR) among the examined groups, it was identified that the eGFR exhibited a significant correlation with CL_R_ for metabolite M4 (Supplementary Table S2; Figure 3). In the case of total and unbound ciprofol, C_max,u_ appeared to be linearly associated with sex, while AUC_0-inf_ or AUC_0-inf,u_ was significantly associated with weight, as indicated in Supplementary Table S2.

### 3.3 Pharmacodynamics

**FIGURE 4:**
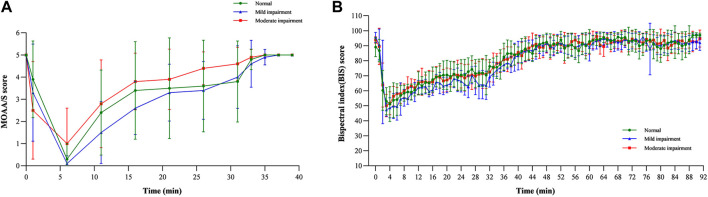
Pharmacodynamic parameter curves for **(A)** MOAA/S score–time and **(B)** BIS value–time.

The results revealed that the MOAA/S score–time curves were similar across the three groups, consistent with the PK profiles (Figure 4A). All three groups experienced deep sedation (MOAA/S 0–1) 6 min after infusion and regained consciousness immediately after infusion (Pastis et al., 2022). The median recovery time for the normal renal function group was 0.00 min, that for the mild renal impairment group was 1.08 min, and that for the moderate renal impairment group was 0.00 min, suggesting that the fluctuations in the recovery time were not significant.

Mean BIS–time profiles and parameters are shown in Figure 4B; Table 4, respectively. The BIS values decreased rapidly following the start of the infusion and reached their lowest levels (ranging from 41.13 to 47.63) at median times of 4.00, 3.48, and 3.00 min post-infusion for the normal function, mild renal impairment, and moderate renal impairment groups, respectively. The mean BIS corresponding to a 3–4 MOAA/S score, the desired sedation depth (Yamada et al., 2022), was 69. BIS_peak_ and BIS AUC_0-t_ were similar across the three groups.

**TABLE 4:** Bispectral index (BIS) parameters in participants with normal and impaired renal function.

Parameter	Renal function group		
	Normal (*n* = 8)	Mild (*n* = 8)	Moderate (*n* = 8)
BIS_peak_	47.63 (10.183)	41.13 (5.515)	47.63 (6.323)
BIS AUC_0-t_	7,445.44 (303.404)	7,270.08 (323.113)	7,448.91 (302.779)
T_BISpeak(min)_	4.00 (2.00, 31.00)	3.48 (1.98, 7.00)	3.00 (2.00, 4.02)

Data are means (SD) for all except T_BISpeak_, which is the median (range).

BIS_peak_, BIS peak value (the lowest BIS value); BIS AUC_0-t_, area under the BIS curve from zero to 1 h after the end of administration; T_BISpeak_, time to BIS peak.

### 3.4 Safety

In the current study, 14 participants (58.3%) encountered a total of 19 AEs, as detailed in Table 5. The trial documented four cases of respiratory adverse events, with one occurring in the mild impairment group and three in the moderate impairment group. This suggests that there may be a potential association between the severity of renal injury and the increased incidence of adverse respiratory reactions. Throughout the entire duration of the trial, no occurrences of participant withdrawal due to adverse effects, severe adverse reactions, or fatalities were documented.

**TABLE 5:** Statistics of adverse events and adverse drug reactions.

System organ class preferred term	Renal function group [N (%), E]		
	Normal (*n* = 8)	Mild (*n* = 8)	Moderate (*n* = 8)
Overall AEs	4 (50%), 5	5 (62.5%), 6	5 (62.5%), 8
Injection site pain	0 (0), 0	1 (12.5%), 1	1 (12.5%), 1
Bradycardia	1 (12.5%), 1	1 (12.5%), 1	0 (0), 0
Electrocardiogram abnormal	0 (0), 0	2 (25%), 2	0 (0), 0
Apnea	0 (0), 0	1 (12.5%), 1	2 (25%), 2
Respiratory depression	0 (0), 0	0 (0), 0	1 (12.5%), 1
Dizziness	0 (0), 0	0 (0), 0	1 (12.5%), 1
Blood pressure increased	1 (12.5%), 1	1 (12.5%), 1	0 (0), 0
Hypotension	1 (12.5%), 1	0 (0), 0	0 (0), 0
Protein present in urine	1 (12.5%), 1	0 (0), 0	0 (0), 0
Facial paralysis	1 (12.5%), 1	0 (0), 0	0 (0), 0
Puncture site swelling	0 (0), 0	0 (0), 0	1 (12.5%), 1
Vessel puncture site ecchymosis	0 (0), 0	0 (0), 0	2 (25%), 2
Drug-related AEs	3 (37.5%), 3	2 (25%), 3	5 (62.5%), 5
Injection site pain	0 (0), 0	1 (12.5%), 1	1 (12.5%), 1
Bradycardia	1 (12.5%), 1	1 (12.5%), 1	0 (0), 0
Apnea	0 (0), 0	1 (12.5%), 1	2 (25%), 2
Respiratory depression	0 (0), 0	0 (0), 0	1 (12.5%), 1
Dizziness	0 (0), 0	0 (0), 0	1 (12.5%), 1
Hypotension	1 (12.5%), 1	0 (0), 0	0 (0), 0
Facial paralysis	1 (12.5%), 1	0 (0), 0	0 (0), 0

E, number of AEs; N, number of subjects with AEs.

**FIGURE 5:**
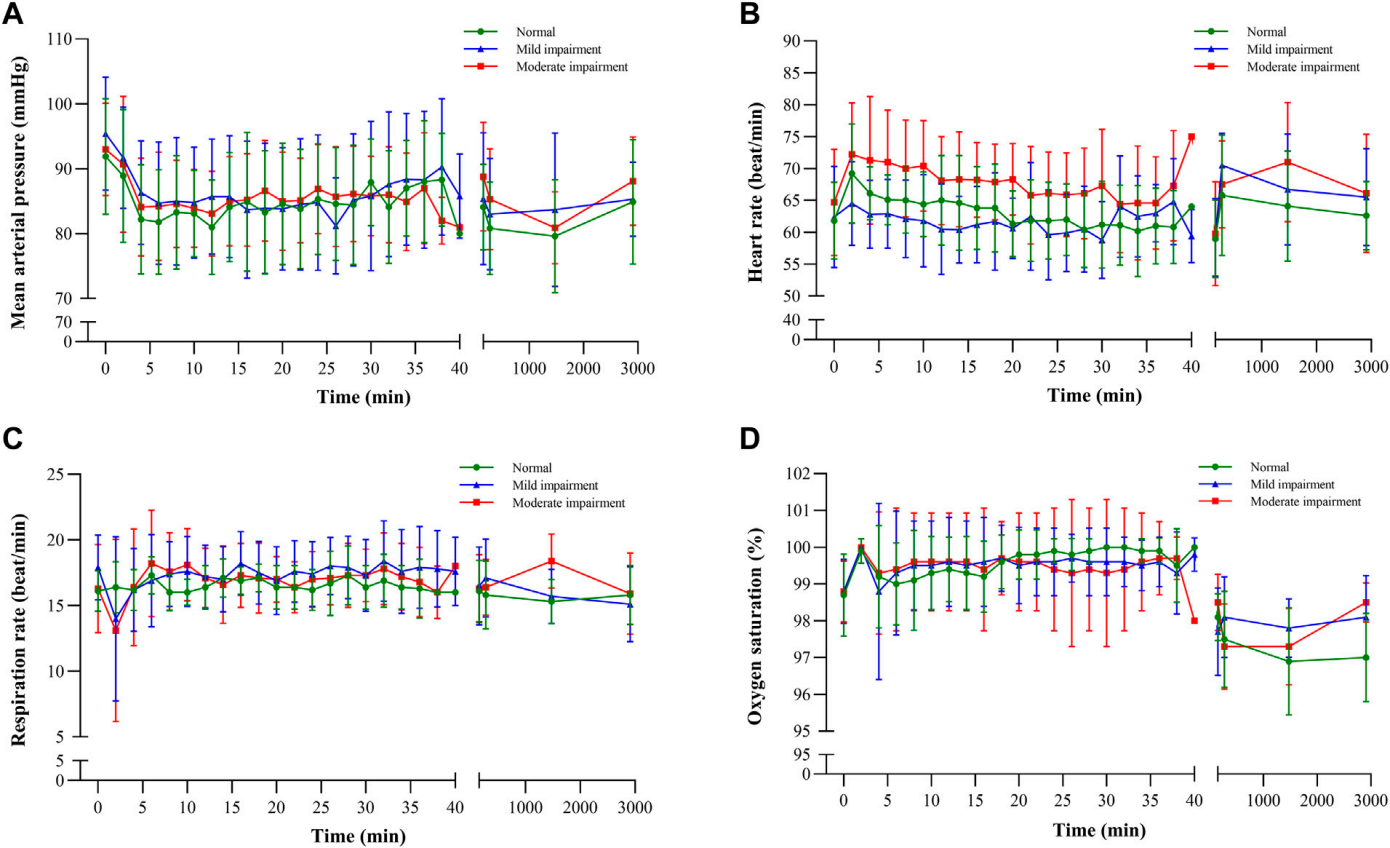
Vital sign–time curves: **(A)** mean arterial pressure, **(B)** heart rate, **(C)** respiration rate, and **(D)** oxygen saturation.

Vital signs, which included diastolic and systolic blood pressures, mean arterial pressure, oxygen saturation, and respiratory and cardiac rates, exhibited a consistent pattern of change (Figure 5), with fluctuations remaining within a 20% range. These observations suggest that ciprofol had minimal impact on hemodynamic stability, signifying its favorable safety profile.

## 4 Discussion

This study is the first to investigate the impact of varying degrees of renal impairment on the PK, PD, and safety of ciprofol, aiming to provide insights for dose adjustment in patients with mild–moderate kidney function impairment. The AUCs and C_max_ of total and unbound ciprofol in plasma for participants with renal impairment were only slightly higher (0.7–1.2-fold) than those in participants with normal renal function. Linear regression analysis revealed that PK parameters for ciprofol did not exhibit a significant correlation with the eGFR. However, given the relatively small sample size and larger variability (geometric % coefficient of variation), the reason behind the observed difference should be further investigated. It is crucial to acknowledge that the variation in C_max_ and AUC between healthy individuals and patients with renal impairment may be influenced by differences in infusion rates or distribution volume. Additionally, variations in C_max,u_ and AUC_u_ could also be linked to variances in protein binding (Celestin and Musteata, 2021). Ciprofol is a drug with a substantial affinity for binding to proteins, primarily associating with serum albumin. It has been documented that the level of albumin tends to decrease in various types of renal diseases (Garrido et al., 1994). However, our study found that with the decrease in renal function, protein binding increased. This indicates that other proteins, including lipoproteins, may have a role in the protein binding of ciprofol (Zamacona et al., 1997; Zamacona et al., 1998).

The AUC_0-inf_ of M4 showed a 1.3–2.1-fold higher concentration in participants with mild and moderate kidney impairment, respectively, compared to participants with normal renal function. The increase in AUC_0-inf_ with the severity of renal impairment is consistent with its renal clearance. This is not surprising as M4 is primarily eliminated by kidneys. However, as M4 has been shown to have no excitatory effects on GABAA receptor-mediated cellular currents (Bian et al., 2021), it may not have a clinically relevant effect on the PD of ciprofol.

UGT1A9 serves as the principal enzyme responsible for the conversion of ciprofol into M4 and is acknowledged to exhibit polymorphic characteristics. Genetic polymorphisms within the UGT1A9 gene have been documented in association with the PK and/or toxicity of various pharmaceutical agents, including propofol, fluoroquinolone, and mycophenolic acid (Lévesque et al., 2007; Kansaku et al., 2011; Annisa et al., 2022). Nevertheless, no investigations to date have explored the potential correlations between UGT1A9 genetic polymorphisms and the PK and/or PD of ciprofol. Consequently, further investigations are imperative to ascertain the plausible impact of these genetic variations on ciprofol’s PK and/or PD.

The temporal profiles of the MOAA/S score and BIS value for PD were similar among the various renal impairment groups, indicating that adjusting ciprofol dosages based on renal function may not be warranted. A significant correlation between ciprofol exposure and parameters such as recovery time, BIS AUC_0-t_, BIS_peak_, and T_BISpeak_ has been observed, suggesting the potential utility of ciprofol exposure as a predictor of therapeutic efficacy. Concerning safety, there was an increased incidence of respiratory adverse reactions in the renal function impaired group, relative to the normal renal function group. Such adverse events are well-recognized in the context of anesthesia administration (Gan, 2006). Diligent monitoring by airway management experts is essential to ensure patient safety (Dinis-Oliveira, 2018). Overall, ciprofol demonstrated favorable tolerability, an acceptable safety profile, and stable patient hemodynamics.

Regrettably, this study did not include patients with severe renal impairment (eGFR: 15–29 mL/min/1.73 m^2^), individuals with end-stage renal disease (ESRD) (eGFR<15 mL/min/1.73 m^2^), or those undergoing dialysis. Notably, ESRD patients may experience gastrointestinal absorption alterations, changes in distribution volumes, shifts in protein binding, and variations in drug metabolic clearance. Patients with ERSD may require higher propofol induction doses (Goyal et al., 2002). The possible causes of high propofol doses in individuals with renal dysfunction include hyperdynamic circulation brought on by anemia. de Gasperi et al. (1996) also found lower plasma propofol concentrations in patients with severe renal impairment, attributed to increased phenol-induced glucuronosyltransferase activity, enhanced glucuronic acid binding, and accelerated hepatic biotransformation in patients with renal dysfunction patients. Nonetheless, clinical data on ciprofol in patients with severe renal impairment and ESRD receiving ciprofol remain unexplored, thus warranting cautious usage in this patient subgroup.

## 5 Conclusion

In summary, patients with mild (eGFR: 60–89 mL/min/1.73 m^2^) to moderate (eGFR: 30–59 mL/min/1.73 m^2^) renal function impairment displayed no clinically significant alterations in the PK and PD of ciprofol. Moreover, ciprofol exhibited favorable tolerance and an acceptable safety profile across all renal function groups. Consequently, the adjustment of ciprofol dosages is deemed unnecessary for individuals with mild to moderately impaired renal function based on the aforementioned findings.

## Data Availability

The original contributions presented in the study are included in the article/Supplementary Material; further inquiries can be directed to the corresponding authors.
