# Supplementary material for: Pharmacokinetics, pharmacodynamics, and safety of ciprofol emulsion in Chinese subjects with normal or impaired renal function
Source: Front Pharmacol. 2023 Nov 23;14:1260599. doi: 10.3389/fphar.2023.1260599 (PMC10704090; doi:10.3389/fphar.2023.1260599)
Supplement: Supplementary file 1 [file Table1.docx]

**Pharmacokinetics, Pharmacodynamics, and Safety of Ciprofol Emulsion in Chinese Subjects with Normal or Impaired Renal Function**

**Jun Tao^1,2,#^, Shuaibing Liu^1,2,#^, Ying Ying Zhao^3^, Lei Qi^1^, Pangke Yan^4^, Nan Wu^4^, Xiao Liu^4^, Dongwei Liu^5*^, Xin Tian^1,2,*^**

Supplementary Table 1. Responsiveness Scores of the Modified Observer’s Assessment of Alertness and Sedation Scale

| Score | Responsiveness |
| --- | --- |
| 5 | Responds readily to demand in normal tone |
| 4 | Lethargic response to demand in normal tone |
| 3 | Responds only after loud and/or repeated demand |
| 2 | Responds only after mild prodding or shaking |
| 1 | Responds only after painful trapezius squeeze |
| 0 | No response after painful trapezius squeeze |

Supplementary Table 2. Multiple regression analysis between clinical variables and PK parameters. The correlation was determined by multiple regression analysis, using PK parameters as the dependent variable and clinical variables as independent variables

| Parameter |  | Estimate | Standard error | 95% confidence interval | P value |
| --- | --- | --- | --- | --- | --- |
| C_max_ | Age | -106.8 | 61.97 | -236.5 to 22.94 | 0.1011 |
|  | Sex | 1964 | 1128 | -395.9 to 4324 | 0.0977 |
|  | Bodyweight | 58.78 | 65.16 | -77.61 to 195.2 | 0.3783 |
|  | eGFR | -165 | 523.5 | -1261 to 930.7 | 0.7561 |
| AUC_0-inf_ | Age | -4.198 | 3.166 | -10.82 to 2.429 | 0.2006 |
|  | Sex | 18.84 | 57.61 | -101.7 to 139.4 | 0.7473 |
|  | Bodyweight | 8.568 | 3.329 | 1.600 to 15.54 | 0.0186 |
|  | eGFR | 16.01 | 26.75 | -39.97 to 71.99 | 0.5565 |
| CL | Age | 0.4374 | 0.3702 | -0.3375 to 1.212 | 0.2520 |
|  | Sex | -1.862 | 6.737 | -15.96 to 12.24 | 0.7852 |
|  | Bodyweight | 0.06926 | 0.3893 | -0.7456 to 0.8841 | 0.8607 |
|  | eGFR | -1.728 | 3.128 | -8.274 to 4.818 | 0.5871 |
| C_max, u_ | Age | -0.4706 | 0.5169 | -1.552 to 0.6113 | 0.3740 |
|  | Sex | 21.51 | 9.405 | 1.823 to 41.19 | 0.0339 |
|  | Bodyweight | 0.7746 | 0.5435 | -0.3630 to 1.912 | 0.1703 |
|  | eGFR | -5.535 | 4.366 | -14.67 to 3.604 | 0.2203 |
| AUC_0-inf,u_ | Age | 0.002598 | 0.02912 | -0.05835 to 0.06355 | 0.9298 |
|  | Sex | 0.5311 | 0.5299 | -0.5779 to 1.640 | 0.3288 |
|  | Bodyweight | 0.09771 | 0.03062 | 0.03363 to 0.1618 | 0.0048 |
|  | eGFR | -0.2702 | 0.2460 | -0.7851 to 0.2447 | 0.2858 |
| CL_u_ | Age | -3.515 | 48.34 | -104.7 to 97.66 | 0.9428 |
|  | Sex | -691.8 | 879.6 | -2533 to 1149 | 0.4412 |
|  | Bodyweight | -21.69 | 50.83 | -128.1 to 84.69 | 0.6743 |
|  | eGFR | 563.6 | 408.3 | -291.1 to 1418 | 0.1836 |
| CL_R_ | Age | -0.006815 | 0.02093 | -0.05062 to 0.03699 | 0.7483 |
|  | Sex | 0.1789 | 0.3808 | -0.6181 to 0.9760 | 0.6438 |
|  | Bodyweight | 0.04308 | 0.02201 | -0.002984 to 0.08914 | 0.0652 |
|  | eGFR | -1.052 | 0.1768 | -1.422 to -0.6824 | <0.0001 |

p < 0.05 was considered statistically significant.

eGFR: the estimated glomerular filtration rate; C_max_: maximum observed concentration; AUC_0-inf_: area under the curve from zero to infinity time; CL: total clearance; u: free fraction; CL_R_: renal clearance.
